# Development and validation of machine learning models to identify high-risk surgical patients using automatically curated electronic health record data (Pythia): A retrospective, single-site study

**DOI:** 10.1371/journal.pmed.1002701

**Published:** 2018-11-27

**Authors:** Kristin M. Corey, Sehj Kashyap, Elizabeth Lorenzi, Sandhya A. Lagoo-Deenadayalan, Katherine Heller, Krista Whalen, Suresh Balu, Mitchell T. Heflin, Shelley R. McDonald, Madhav Swaminathan, Mark Sendak

**Affiliations:** 1 Duke Institute for Health Innovation, Durham, North Carolina, United States of America; 2 Department of Statistical Sciences, Duke University, Durham, North Carolina, United States of America; 3 Department of Surgery, Duke University, Durham, North Carolina, United States of America; 4 Division of Geriatrics, Department of Medicine, Duke University, Durham, North Carolina, United States of America; 5 Department of Anesthesiology, Duke University, Durham, North Carolina, United States of America; Barts and the London School of Medicine & Dentistry Queen Mary University of London, UNITED KINGDOM

## Abstract

**Background:**

Pythia is an automated, clinically curated surgical data pipeline and repository housing all surgical patient electronic health record (EHR) data from a large, quaternary, multisite health institute for data science initiatives. In an effort to better identify high-risk surgical patients from complex data, a machine learning project trained on Pythia was built to predict postoperative complication risk.

**Methods and findings:**

A curated data repository of surgical outcomes was created using automated SQL and R code that extracted and processed patient clinical and surgical data across 37 million clinical encounters from the EHRs. A total of 194 clinical features including patient demographics (e.g., age, sex, race), smoking status, medications, comorbidities, procedure information, and proxies for surgical complexity were constructed and aggregated. A cohort of 66,370 patients that had undergone 99,755 invasive procedural encounters between January 1, 2014, and January 31, 2017, was studied further for the purpose of predicting postoperative complications. The average complication and 30-day postoperative mortality rates of this cohort were 16.0% and 0.51%, respectively. Least absolute shrinkage and selection operator (lasso) penalized logistic regression, random forest models, and extreme gradient boosted decision trees were trained on this surgical cohort with cross-validation on 14 specific postoperative outcome groupings. Resulting models had area under the receiver operator characteristic curve (AUC) values ranging between 0.747 and 0.924, calculated on an out-of-sample test set from the last 5 months of data. Lasso penalized regression was identified as a high-performing model, providing clinically interpretable actionable insights. Highest and lowest performing lasso models predicted postoperative shock and genitourinary outcomes with AUCs of 0.924 (95% CI: 0.901, 0.946) and 0.780 (95% CI: 0.752, 0.810), respectively. A calculator requiring input of 9 data fields was created to produce a risk assessment for the 14 groupings of postoperative outcomes. A high-risk threshold (15% risk of any complication) was determined to identify high-risk surgical patients. The model sensitivity was 76%, with a specificity of 76%. Compared to heuristics that identify high-risk patients developed by clinical experts and the ACS NSQIP calculator, this tool performed superiorly, providing an improved approach for clinicians to estimate postoperative risk for patients. Limitations of this study include the missingness of data that were removed for analysis.

**Conclusions:**

Extracting and curating a large, local institution’s EHR data for machine learning purposes resulted in models with strong predictive performance. These models can be used in clinical settings as decision support tools for identification of high-risk patients as well as patient evaluation and care management. Further work is necessary to evaluate the impact of the Pythia risk calculator within the clinical workflow on postoperative outcomes and to optimize this data flow for future machine learning efforts.

## Abstract 2

Leveraging a single-site surgical EHR data pipeline and repository, Kristin Corey and colleagues present a machine learning-based detection of high-risk surgical patients at their institution.

## Author summary

**Why was this study done?:**

- Most postoperative complication risk prediction models use American College of Surgeons (ACS) National Surgical Quality Improvement Program (NSQIP) data.
- Few published postoperative risk prediction models using electronic health record (EHR) data exist.
- Creating and updating manual datasets such as ACS NSQIP are intensive processes with regards to time, labor, and cost.

**What did the researchers do and find?:**

- A curated data repository of postoperative outcomes was created that extracted and processed patient clinical and surgical data across 37 million clinical encounters in EHRs into 194 clinical features.
- Machine learning models were built off this dataset to predict risk of postoperative complications. Models were able to classify patients at high risk of postoperative complication with high sensitivity and specificity.
- An online calculator requiring input of 9 data fields was created to produce a risk assessment within the clinic environment.

**What do these findings mean?:**

- Machine leaning models built off an automatically extracted and curated EHR surgical dataset have strong predictive performance for detecting surgical complications.
- The long-term cost of updating this surgical dataset is lower than that of manually updated datasets.

## Introduction

Complications arise in 15% of all US surgical procedures performed, with high-risk surgeries having complications in up to 50% of cases [1]. In addition to worsening quality of life, surgical complications in the US cost on average over $11,000 per major 30-day complication [2]. With an estimated 19 million surgeries performed each year [3], the total cost of surgical complications per year in the US is approximately $31.35 billion. In response, efforts to enhance preoperative and perioperative support for high-risk and high-cost patients are increasing nationwide [4]. Targeted preoperative intervention clinics for high-risk individuals have been shown to improve 30-day postoperative outcomes at a multisite, quaternary health center [5]. However, the task of identifying these patients within a preoperative setting is challenged by difficulties in timely access to pertinent patient care data and lack of robust predictive models.

The most widespread pre-surgical high-risk patient identification program is the National Surgical Quality Improvement Program (NSQIP) calculator developed by the American College of Surgeons (ACS). This online risk prediction calculator represents national surgical data from 393 different institutions [6]. It has been shown that predictive models built from nationally derived databases have limited local accuracy due to an average effect derived from aggregating data from many different institutions, populations, and regions. Cologne et al. demonstrated that NSQIP postoperative risk predictions differed significantly in terms of length of stay, surgical site infections, and major complications from actual rates at a single institution [7]. Moreover, Etzioni et al. and Osborne et al. demonstrated that enrollment in and feedback from NSQIP are not associated with improved postoperative outcomes or lower Medicare payments among surgical patients [8,9]. This indicates the need for institution-specific improvement efforts driven by highly curated institution-specific data.

The aggregation of health data within each local institution’s electronic health records (EHRs) serves as fertile ground for machine learning to transform healthcare. Machine learning models utilizing EHR data to predict in-hospital length of stay and mortality as well as postoperative complications can be more accurate than prediction models built from manually collected data [10–12]. However, despite the maturation of methodological approaches to working with health data, there has been limited impact on provider productivity and patient outcomes [13]. Current health information technology infrastructure does not facilitate rapid transmission of data between EHRs and model applications. Furthermore, building technologies that integrate with current EHR systems requires significant financial investment [14,15].

The primary aim of this study was to demonstrate an initial use case of machine learning leveraging an institute-specific surgical data pipeline and repository derived from EHRs, Pythia, to identify patients at high risk of post-surgical complications. Pythia was built as part of an innovation initiative to efficiently curate high-volume, high-quality data to monitor surgical care and outcomes. Although EHR data can be inaccurate or incomplete [16], models that are developed and validated on local, structured data in EHRs are best positioned for deployment to support clinical workflows [17]. Pythia was designed to both promote the development of machine learning models and bridge the translational gap to enable rapid deployment of validated models. The secondary aim of this study was to describe machine learning model design decisions that supported clinical interpretability and rapid development of a decision support tool to be used within the preoperative clinic workflow. This decision support tool enables surgeons and referring clinicians to identify high-risk patients who may require targeted assessments and optimization as part of their preoperative care.

## Methods

This project was approved by the Duke Institutional Review Board (Pro00081702), with waiver of informed consent. This was a single-center, retrospective study at Duke University Health System (DUHS), a large, quaternary, multisite hospital system that had 68,000 inpatient stays and more than 2 million outpatient visits in 2017 [18]. This study is reported as per the Transparent Reporting of a Multivariable Prediction Model for Individual Prognosis or Diagnosis (TRIPOD) guidelines (S1 Checklist) [19].

### Training dataset for models

**Fig 1:**
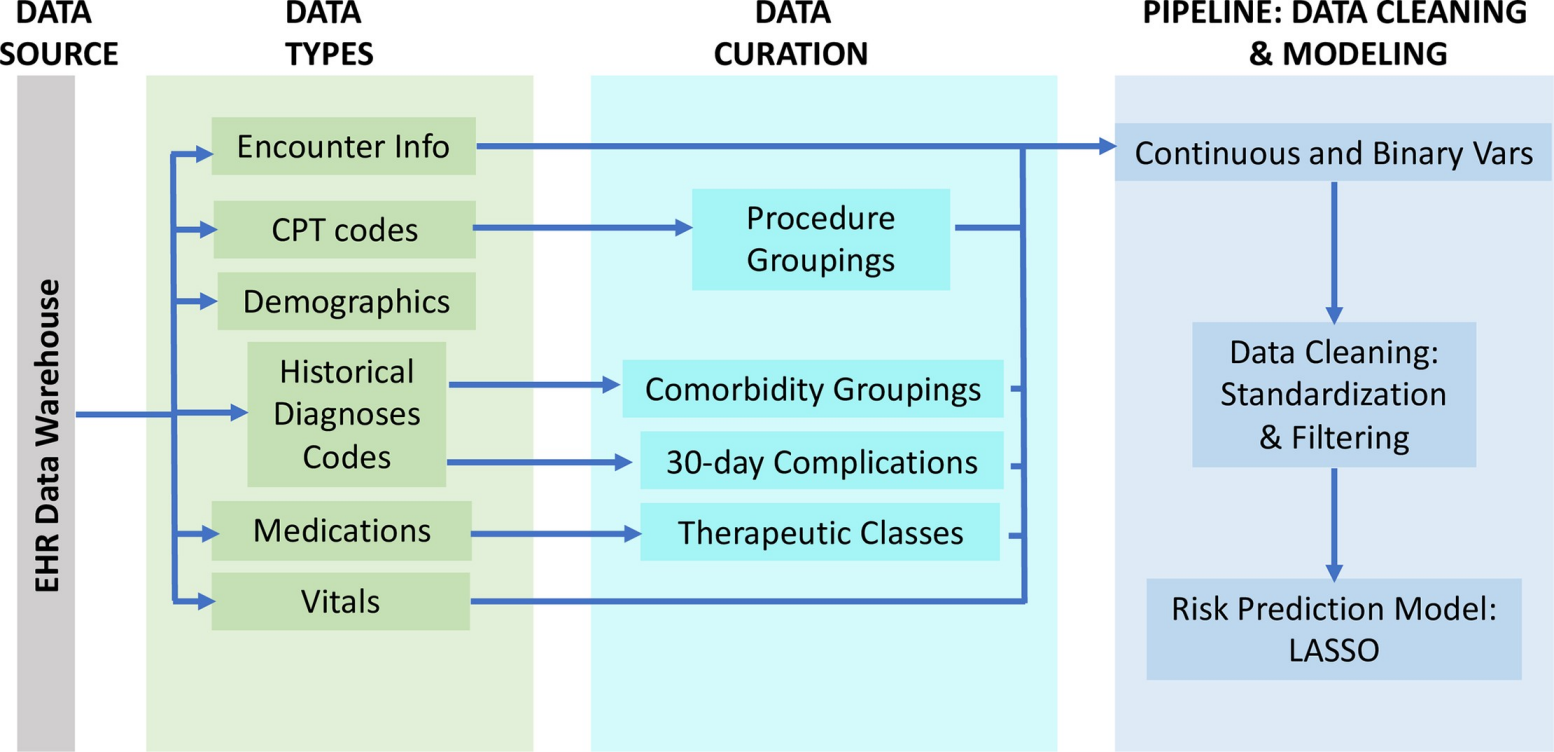
SQL and R code were written to extract, clean, and curate patient data from the electronic health record (EHR) data warehouse. Extracted raw data elements included inpatient and outpatient encounter information, Current Procedural Terminology (CPT) codes, patient demographics, International Classification of Diseases (ICD) codes, medications, and vitals. These elements were curated into clinical features to feed into a cleaning data pipeline to populate Pythia’s data repository. Vars, variables.

A cohort of 163,599 patients was identified in the DUHS EHR system who had undergone any surgical procedure between June 1, 2012, and May 31, 2017. Clinical patient data were extracted from the EHR Oracle database across all inpatient and outpatient encounters using SQL queries. Outpatient and inpatient medications, vital signs, diagnoses, procedures, and orders were extracted across 37,195,164 inpatient and outpatient encounters. Patient demographic and social history data including age, sex, BMI, race, and smoking status were also extracted (Fig 1).

A cohort of 90,145 patients that had undergone 145,604 invasive procedures between January 1, 2014, and January 31, 2017, was identified to develop machine learning models to predict post-surgical complications. Patients under the age of 18 years were excluded from the cohort. Encounters with a CPT code included in the Surgery Flag Software [20] were defined as invasive procedures and included in the cohort. All CPT codes for invasive procedures were grouped into 128 procedure groupings (S1 Table). Predictor variables for the models included comorbidities, number of CPT codes recorded during the invasive procedure, outpatient medications, and demographics. Patient comorbidities were identified by surveilling all ICD codes within 1 year preceding the date of the procedure. These diagnosis codes were then classified into 29 binary comorbidity groupings (S1 Table) as defined by the Elixhauser Comorbidity Index [21]. Patients’ active outpatient medications recorded during medication reconciliation at preoperative visits were classified into 15 therapeutic binary indicator groupings (S1 Table), along with a separate feature that counted the total number of active medications. Surgical complications were defined by diagnosis codes occurring within 30 days following the surgical procedure. In total, 271 diagnosis codes (S2 Table) were grouped into 12 groupings that aligned with prior studies evaluating post-surgical complications [5]. A composite variable, “any complication,” for each procedure was created by aggregating across all 12 complication groupings, and mortality was identified as death occurring within 30 days of the index procedure date. Mortality was captured in the EHRs during encounters (for in-hospital deaths) or uploaded from the Social Security Death Index (for out-of-hospital deaths). In total, 99,755 encounters had complete information on the set of 194 predictors and 14 postoperative outcome groupings, including any complication and 30-day mortality. Encounters missing EHR data were deemed not missing at random and were therefore excluded from the model development cohort. This cohort was used for training, validating, and testing model prediction algorithms. SQL and R code were subsequently written to extract, clean, and curate patient data from the EHRs to the surgical data repository. Efforts to automate this process are currently underway.

### Machine learning methods

Due to the high dimensionality of the input features and the large sample size, machine learning methods were used to model the likelihood of post-surgical complications. Least absolute shrinkage and selection operator (lasso) penalized logistic regression [22], random forest [23], and extreme gradient boosted decision tree [24] models were trained on 88,255 surgical encounters from the Pythia data repository for each of the 14 postoperative outcomes (13 outcome groupings and 30-day mortality). Lasso is an *l*_1_-penalized regression method that performs both regularization and variable selection, which results in a regression solution with improved interpretability and prediction accuracy compared to other regression approaches. Random forests are an ensemble learning method in which multiple decision trees are constructed and averaged to form a solution that is resistant to overfitting to the training data. Lastly, boosted decision trees are another ensemble decision tree approach that aims to optimize a differential loss function; in our case, the loss function is the area under the receiver operator characteristic curve (AUC). The latest set of 11,500 (11.5%) encounters, from October 1, 2016, to January 31, 2017, was excluded from the complete set of 99,755 encounters, for validation testing. This was done in order to provide estimates of how the models would perform if put into operations currently within our local setting.

Ten-fold cross-validation was used within the training set to train lasso models, using the R package glmnet [25] to find the optimal shrinkage hyperparameter for each of the 14 outcomes. Random forests were trained using the R package randomforest [26]. The number of trees was set to 500, and the number of candidate splits was determined using cross-validation across a range of possible values. Lastly, extreme gradient boosted decision tree models were trained using the R package XGboost [27], where the learning rate, eta, and depth of trees were chosen by cross-validation across possible ranges of values. The chosen hyperparameters were cross-validated for each individual outcome across these 3 model types.

Lasso penalized logistic regression, random forest models, and extreme gradient boosted decision trees were chosen specifically due to their ability to provide variable importance and interpretability. By providing model users with additional information about predictor weights, clinicians can glean insights into potential patient risk mitigation strategies. During experiments, elastic net penalized logistic regression models were built as well, but their performance was almost identical to that of the lasso models and they were therefore omitted. Furthermore, by comparing different approaches, clinician users can understand how different types of machine learning models perform on large complex EHR patient data.

Superior models were chosen based on predictive performance measured by AUC, sensitivity, and specificity, with a focus on clinical interpretability. After model selection, an online calculator was built using R Shiny [28] for use in the clinic. The calculator was organized into 3 sections requiring patient information: (1) procedure details, (2) demographic and social history, and (3) patient comorbidities and outpatient medications. The calculator ran the selected machine learning models to provide complication risk scores for all 14 outcomes. Complication risks greater than 5% were displayed on the user interface, as requested by clinical partners. A high-risk threshold that maximizes the sum of sensitivity and specificity for the “any complication” outcome was chosen in order to identify high-risk patients requiring further evaluation.

## Results

Table 1 displays summary statistics for all invasive procedures within Pythia and the machine learning model cohort. In the model cohort, 45% of encounters involved male patients, and the average age was 62.1 years. The most common comorbidities were hypertension (47.4%), tumor without metastasis (13.8%), and uncomplicated diabetes (13.4%). The most common outpatient medications were cardiovascular drugs (68.2%), analgesics (40.0%), and antiplatelet drugs (32.8%). Post-surgical complication rates were 16.0% for any complication within 30 days and 0.5% for death within 30 days. Characteristics between the 2 groups were consistently similar. Pythia encounters excluded from the machine learning model cohort were most often missing active outpatient medications.

**Table 1:** Baseline and clinical characteristics of invasive surgical procedures.

Baseline characteristic	Invasive procedures within Pythia (Jan 2014–Jan 2017) (*N* = 145,604)	Machine learning model cohort (*N* = 99,755)
**Patients**	90,145	66,370
**Age (years)**	59.69 (±14.33)	62.10 (±14.36)
**Sex male**	45%	45%
**Procedure codes**		
Total Current Procedural Terminology codes	192,300	131,800
Unique codes	2,664	2,450
Unique procedure classes	128	127
**Race**		
White	104,846 (72.0%)	72,657 (72.8%)
Black or African American	30,798 (21.2%)	21,230 (21.3%)
Asian	2,264 (1.6%)	1253 (1.3%)
2 or more races	1,304 (0.8%)	701 (0.7%)
American Indian or Alaska Native	599 (0.4%)	432 (0.4%)
Other/missing	5,793 (4.0%)	3,482 (3.5%)
**Comorbidities**		
Hypertension	58,592 (40.2%)	47,273 (47.4%)
Solid tumor without metastasis	18,885 (13.0%)	13,799 (13.8%)
Diabetes without chronic complications	16,480 (11.3%)	13,361 (13.4%)
Chronic pulmonary disease	15,661 (10.8%)	11,877 (11.9%)
Obesity	15,542 (10.7%)	11,405 (11.4%)
Deficiency anemias	14,055 (9.7%)	10,103 (10.1%)
Depression	13,188 (9.1%)	10,007 (10.0%)
Hypothyroidism	12,212 (8.4%)	9,264 (9.3%)
Fluid and electrolyte disorders	11,951 (8.2%)	8,608 (8.6%)
Diabetes with chronic complications	11,834 (8.1%)	8,586 (8.6%)
Renal failure	11,744 (8.1%)	8,781 (8.8%)
Other neurological disorders	8,905 (6.1%)	6,452 (6.5%)
Peripheral vascular disease	8,790 (6.0%)	6,593 (6.6%)
Congestive heart failure	7,601 (5.2%)	5,465 (5.5%)
Valvular heart disease	7,143 (4.9%)	5,337 (5.4%)
Psychoses	4,895 (3.4%)	3,694 (3.7%)
Rheumatoid arthritis	4,711 (3.2%)	3,795 (3.8%)
Coagulation deficiency	4,114 (2.8%)	2,872 (2.9%)
Weight loss	3,885 (2.7%)	2,695 (2.7%)
Pulmonary circulation disorders	3,719 (2.6%)	2,585 (2.6%)
Metastatic cancer	3,227 (2.2%)	2,330 (2.3%)
Drug abuse	3,114 (2.1%)	2,331 (2.3%)
Liver disease	3,049 (2.1%)	2,338 (2.3%)
Alcohol abuse	1,633 (1.1%)	1,096 (1.1%)
Blood loss anemias	1,314 (0.9%)	829 (0.8%)
Lymphoma	1,256 (0.9%)	966 (1.0%)
Paralysis	1,156 (0.8%)	790 (0.8%)
HIV or AIDS	318 (0.2%)	246 (0.3%)
Peptic ulcer disease	47 (0.0%)	40 (0.0%)
**Complications within 30 days**		
Any complication	22,554 (15.5%)	15931 (16.0%)
Cardiac	8,439 (5.8%)	6,086 (6.1%)
Genitourinary	3,669 (2.5%)	2,644 (2.7%)
Pulmonary	3,616 (2.5%)	2,420 (2.4%)
Hematological	3,121 (2.1%)	2,198 (2.2%)
Neurological	2,634 (1.8%)	1,770 (1.8%)
Gastrointestinal	2,432 (1.7%)	1,706 (1.7%)
Renal	2,358 (1.6%)	1,678 (1.7%)
Endocrine	2,136 (1.5%)	1,650 (1.7%)
Vascular	2,057 (1.4%)	1,247 (1.3%)
Integumentary	1,930 (1.3%)	1,373 (1.4%)
Sepsis	1,902 (1.3%)	1,302 (1.3%)
Shock	911 (0.6%)	664 (0.7%)
Death	768 (0.5%)	508 (0.5%)
Falls	494 (0.3%)	326 (0.3%)
Inpatient procedures	42,228 (29.0%)	30,426 (30.5%)
**Active medications**		
Cardiovascular	73,640 (68.0%)	68,013 (68.2%)
Analgesics	43,557 (40.2%)	39,946 (40.0%)
Antiplatelet drugs	35,688 (32.9%)	32,702 (32.8%)
Diuretics	26,713 (24.7%)	24,538 (24.6%)
Cardiac drugs	26,214 (24.2%)	24,103 (24.2%)
Central nervous system drugs	24,209 (22.3%)	22,393 (22.4%)
Antihyperglycemics	23,328 (21.5%)	21,331 (21.4%)
Antibiotics	17,974 (16.6%)	16,765 (16.8%)
Hormones	15,516 (14.3%)	14,404 (14.4%)
Anticoagulants	6,519 (6.1%)	5,830 (5.8%)
Antineoplastics	4,305 (4.3%)	3,985 (4.0%)
Antivirals	4,116 (3.8%)	3,803 (3.8%)
Autonomic drugs	3,325 (3.1%)	3,061 (3.1%)
Immunosuppressants	2,821 (2.6%)	2,627 (2.6%)
Anesthetics	2,225 (2.1%)	2,063 (2.1%)
**Average number of active medications**	3.94 (±2.66)	3.93 (±2.65)

Data are given as *n*, percent, *n* (percent), or mean (±SD). Left column shows the summary statistics for the set of all invasive surgical encounters, whereas the right column shows the same statistics for the set of encounters with complete predictor information that was used for the training and testing cohorts. Active outpatient medication percentages are over the non-missing rows (*N* = 108,400).

**Fig 2:**
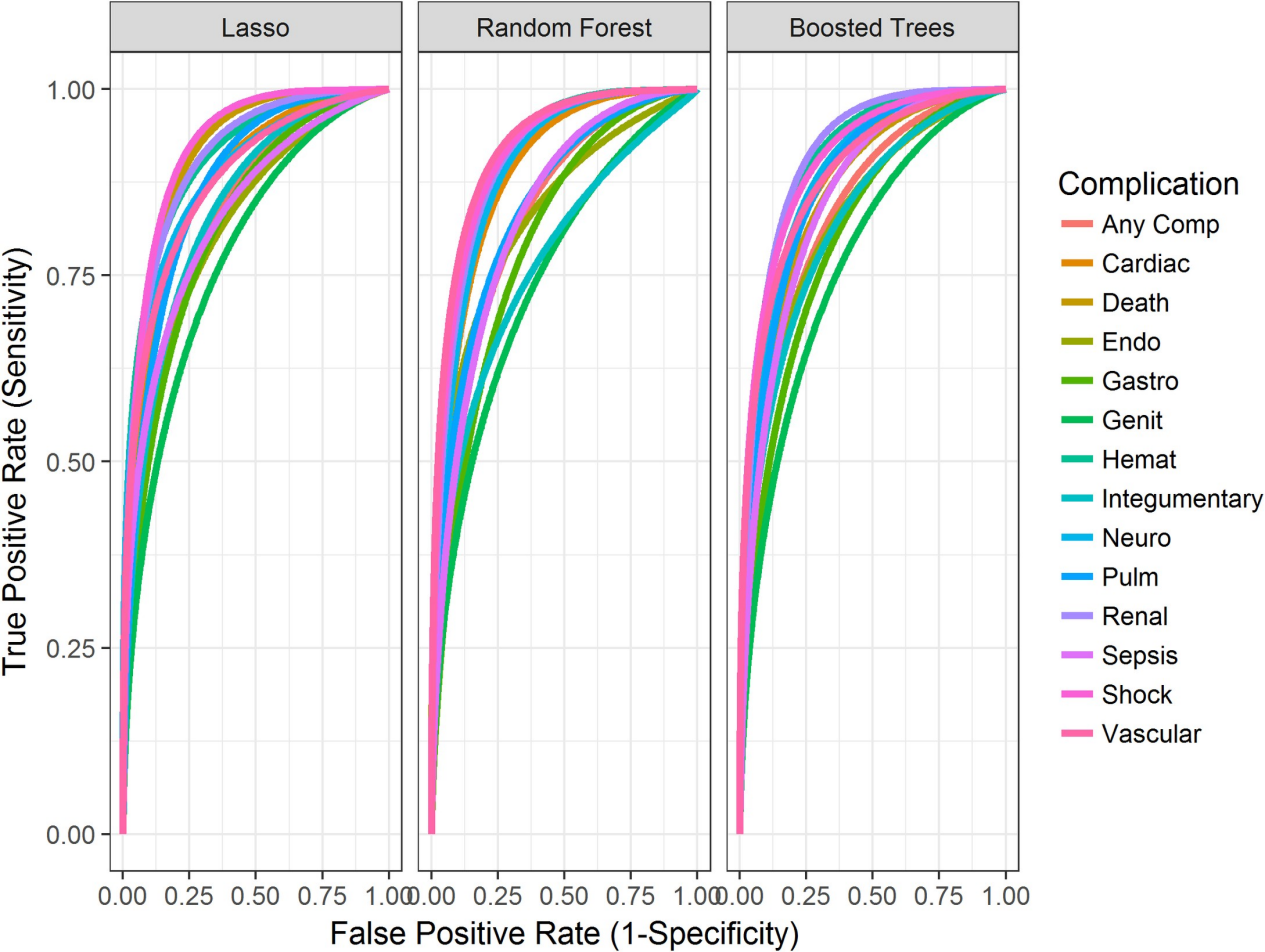
Graphs displaying the resulting receiver operator characteristic curves for each modeling method across all 14 complications. comp, complication; endo, endocrine; gastro, gastrointestinal; genit, genitourinary; hemat, hematological; lasso, least absolute shrinkage and selection operator; neuro, neurological; pulm, pulmonary.

The resulting 42 models (lasso, random forest, and extreme gradient boosted decision trees for 14 outcomes) overall demonstrated strong predictive performance, with AUCs ranging between 0.747 and 0.924 (Table 2) calculated on a non-random, out-of-sample test set of the latest patient encounters from a different time period than the training set data. The size of this test set was 11,500 encounters. However, the lasso penalized logistic regression performed slightly superiorly to the random forest models, with AUCs ranging from 0.747 to 0.903. Lasso and extreme gradient boosted decision tree models performed very similarly. However, lasso outperformed extreme gradient boosted decision trees in 8 outcome models (any complication, 30-day mortality, gastrointestinal, genitourinary, hematological, integumentary, renal, and shock), while extreme gradient boosted decision trees outperformed lasso in 5 of the remaining outcome models (cardiac, endocrine, pulmonary, sepsis, and vascular). Neurological outcome models had the same AUC performance (0.810) in lasso and extreme gradient boosted decision trees. The receiver operator characteristic curves in Fig 2 display the resulting curve for each modeling method across all complications. This visualization confirms that lasso, random forest, and extreme gradient boosted decision trees performed very similarly. These well-established and interpretable models achieved strong performance on structured data that are highly available within our EHR system, paving the way for rapid deployment of an application to impact patient care.

**Table 2:** AUCs and 95% CIs for lasso, random forest, and extreme gradient boosted decision trees for 14 postoperative outcomes.

Outcome	AUC (95% CI)		
	Lasso	Random forest	Extreme gradient boosted decision trees
Any complication	0.836 (0.825, 0.846)	0.829 (0.819, 0.840)	0.835 (0.825, 0.846)
Cardiac	0.880 (0.867, 0.893)	0.879 (0.860, 0.888)	0.883 (0.871, 0.896)
30-day mortality	0.916 (0.883, 0.950)	0.832 (0.761, 0.903)	0.861 (0.805, 0.917)
Endocrine	0.815 (0.779, 0.850)	0.798 (0.760, 0.837)	0.828 (0.795, 0.861)
Gastrointestinal	0.820 (0.790, 0.849)	0.781 (0.749, 0.813)	0.804 (0.774, 0.836)
Genitourinary	0.781 (0.752, 0.810)	0.747 (0.717, 0.777)	0.772 (0.744, 0.801)
Hematological	0.908 (0.890, 0.925)	0.886 (0.865, 0.908)	0.906 (0.889, 0.924)
Integumentary	0.845 (0.813, 0.876)	0.789 (0.749, 0.829)	0.826 (0.791, 0.860)
Neurological	0.890 (0.864, 0.917)	0.884 (0.859, 0.909)	0.890 (0.866, 0.913)
Pulmonary	0.871 (0.851, 0.891)	0.844 (0.817, 0.870)	0.875 (0.854, 0.895)
Renal	0.910 (0.891, 0.930)	0.903 (0.881, 0.925)	0.909 (0.888, 0.930)
Sepsis	0.835 (0.795, 0.875)	0.814 (0.773, 0.855)	0.850 (0.815, 0.885)
Shock	0.924 (0.901, 0.946)	0.864 (0.821, 0.908)	0.904 (0.873, 0.936)
Vascular	0.878 (0.843, 0.913)	0.886 (0.852, 0.921)	0.890 (0.861, 0.920)

AUC, area under the receiver operator characteristic curve; lasso, least absolute shrinkage and selection operator.

**Fig 3:**
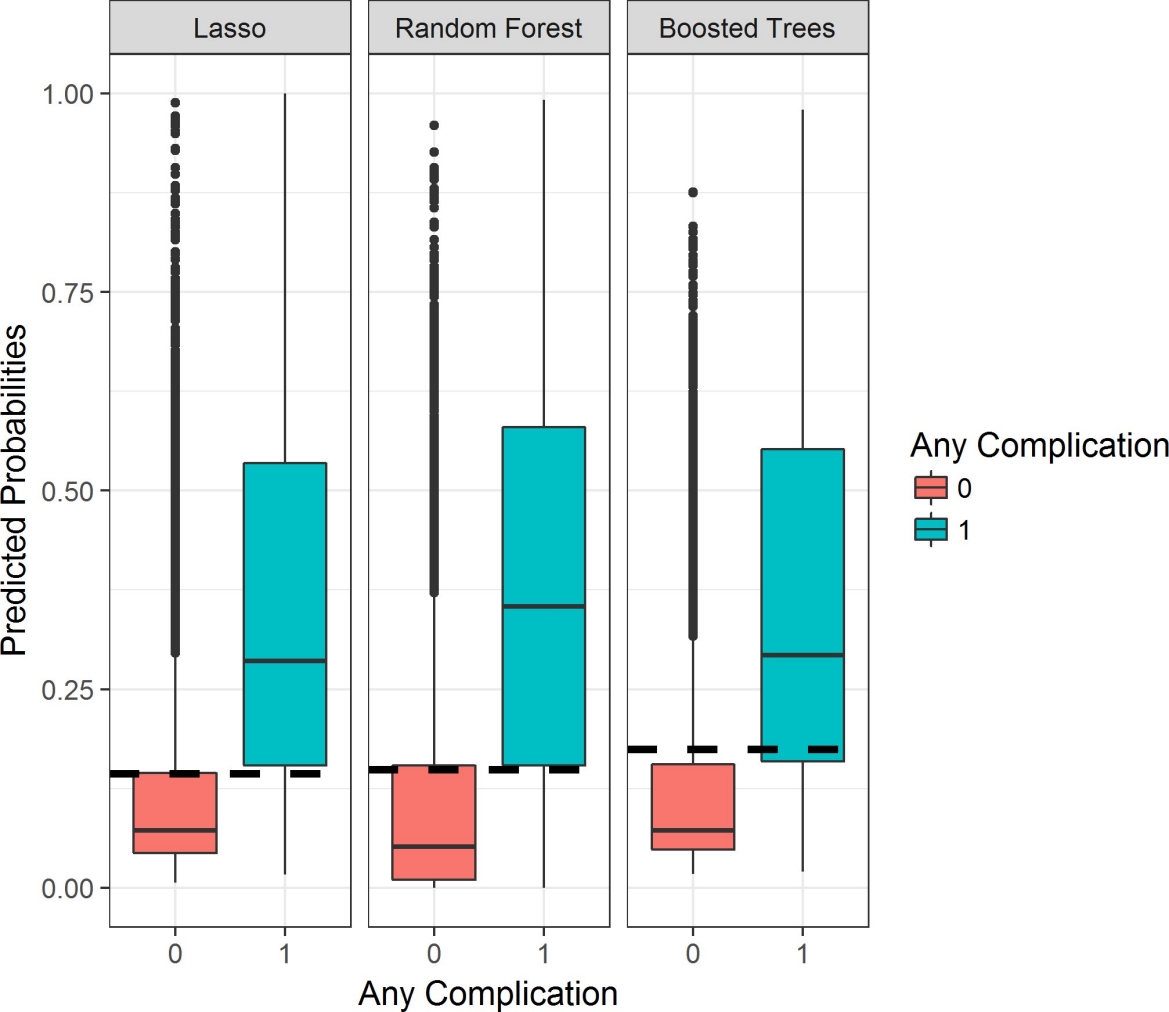
Boxplots displaying the model predicted probabilities for patients who experienced a postoperative complication versus those who did not. Any postoperative complication (category = 1) versus no complications (category = 0). Thresholds (dashed lines) were chosen based on these population delineations. lasso, least absolute shrinkage and selection operator.

High-risk thresholds were determined with the aim of identifying patients needing referrals to targeted perioperative optimization treatment programs. Fig 3 displays predicted probabilities for patients who experienced a postoperative complication and for those who did not for each machine learning model. This plot demonstrates a clear delineation between the 2 populations, with patients experiencing postoperative complications having higher risk predictions. Due to the differences in distributions displayed, the high-risk threshold was purposefully chosen as a percent risk value bordering between the 2 population distributions, resulting in a strong cutoff. Specifically, the thresholds were chosen by maximizing the sum of sensitivity and specificity. The resulting sensitivities and specificities are displayed in Table 3. Under a threshold of 0.142 (14.2% risk of any complication), a sensitivity of 0.775, specificity of 0.749, and positive predictive value (PPV) of 0.362 were achieved with lasso modeling. The resulting sensitivities and specificities were similar across methods. A threshold of 14.9% was chosen for random forest models, and a threshold of 17.4% for extreme gradient decision tree models, resulting in a sensitivity of 0.757 and 0.725, a specificity of 0.744 and 0.792, and a PPV of 0.351 and 0.390, respectively. Consequently, lasso and extreme gradient boosted decision tree models identified a more concentrated group of patients with higher complication rates (36% and 39%) than random forest (35%). Furthermore, lasso and extreme gradient boosted decision tree models have a higher PPV (36.2% and 39.0%) compared to random forest (35.1%), thereby better identifying high-risk patients who then have postoperative complications. However, in order to optimize model performance for healthcare providers by providing clinically interpretable insights regarding risk factors, and identifying a more targeted number of patients, we chose the 14 lasso models to predict complication risk through the online web application for DUHS clinicians. This was done because lasso models allow for better interpretability of which particular health predictors will affect a patient’s risk of postoperative complication, as well as how much each predictor affects the predicted postoperative outcome. Our clinical partners wanted this insight within this decision support tool.

**Table 3:** Resulting threshold, sensitivity, specificity, and PPV for each of the 3 modeling methodologies for the composite outcome, any complication.

Method	Threshold	Sensitivity	Specificity	PPV	High-risk patients (%)
Lasso	0.144	0.775	0.749	0.362	36%
Random forest	0.149	0.757	0.744	0.351	35%
Extreme gradient boosted decision trees	0.174	0.725	0.792	0.390	39%

The percent of high-risk patients selected by each method is also displayed. All results were calculated on the held-out test set of 11,500 encounters.

lasso, least absolute shrinkage and selection operator; PPV, positive predictive value.

**Fig 4:**
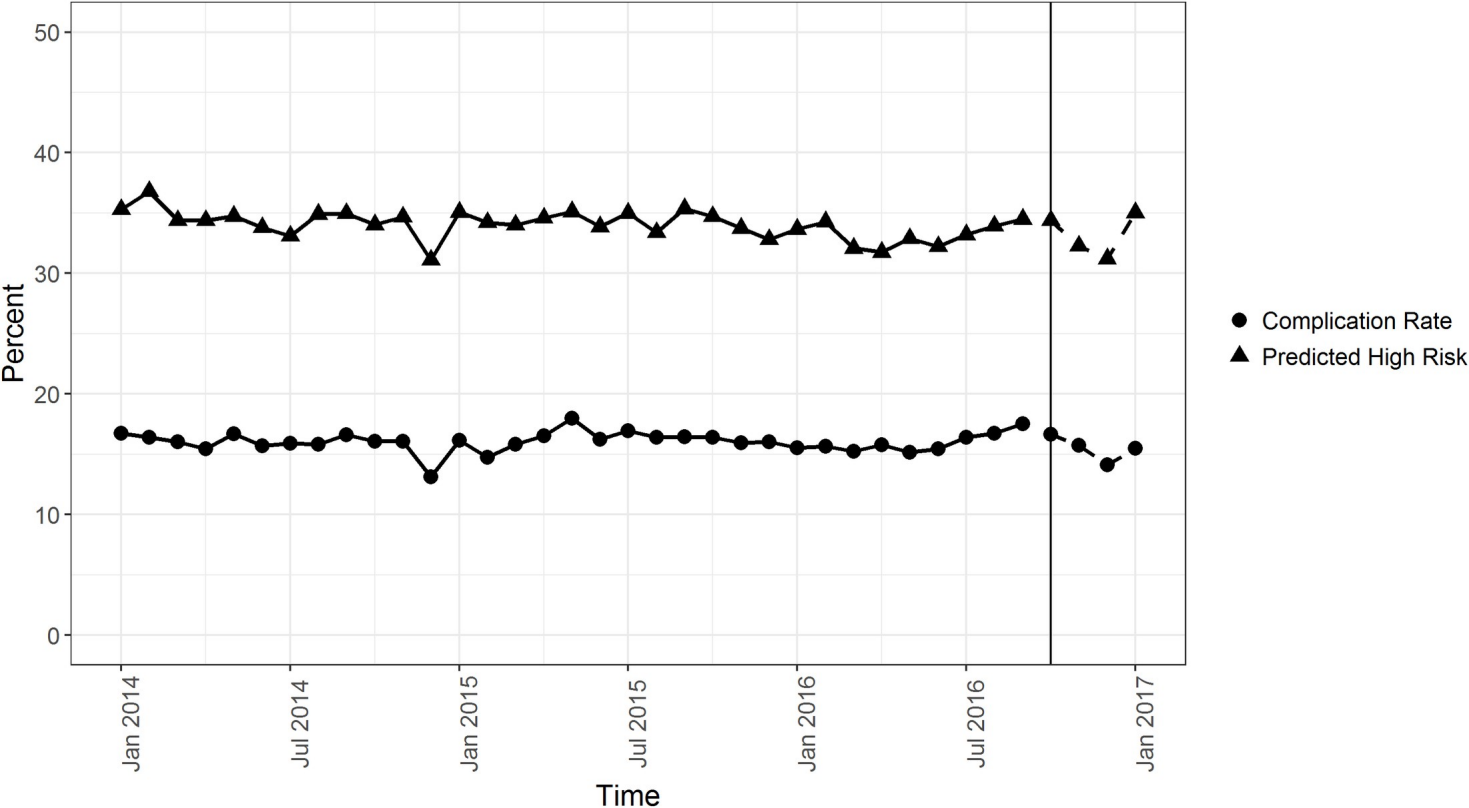
Predicted 30-day complication rate based on lasso model versus observed complication rates. January 2014–September 2016 shows predicted complication rates of the training data, and October 2016–January 2017 displays projected complication rates based on a non-random, out-of-sample test set from a different time period. lasso, least absolute shrinkage and selection operator.

In order to test model stability over time, the observed versus predicted rate of any complication in our data was plotted (Fig 4). Using a high-risk threshold of 14.4% risk of complication, the machine learning models predict that approximately 35% of procedures will result in a complication, while the actual rate of complications is approximately 17% at DUHS over time. This rate difference was intentional, to increase sensitivity to capture more high-risk patients for perioperative optimization. Our clinical and operational partners felt that targeted interventions could comfortably accommodate 35% of patients undergoing invasive procedures. In addition, Fig 4 demonstrates that our model captures underlying patient patterns that occur within our local setting, without information about seasonal or time trends.

In response to requests made by peer reviewers, we further assessed Pythia’s model performance through a local validation analysis by comparing our methods to expert clinical criteria for a geriatric preoperative optimization intervention within our healthcare system. Expert clinical criteria for geriatric high risk included patients taking more than 5 medications or with multiple comorbidities, neurological disorders, or recent weight loss, as previously described [5]. A new test set (*n* = 5,734) was identified using our original test set of all patients on or after October 1, 2016, and filtering for geriatric patients (age > 65 years). Our model identified 1,933 patients with risk scores above our model’s high-risk threshold. In comparison, we identified 3,102 geriatric patients using expert clinical criteria within the same test set. The mean complication rate for high-risk patients identified by our model was 37.99%, while the mean complication rate for patients identified by clinical criteria was 16.55%, indicating that our model identified a more specific high-risk cohort of patients. The sensitivity, specificity, and PPV for each methodology of risk stratification were also determined, with our model having a sensitivity of 77.24%, specificity of 74.92%, and PPV of 37.92% compared to a sensitivity of 73.45%, specificity of 49.74%, and PPV of 22.47% for the expert clinical criteria.

**Fig 5:**
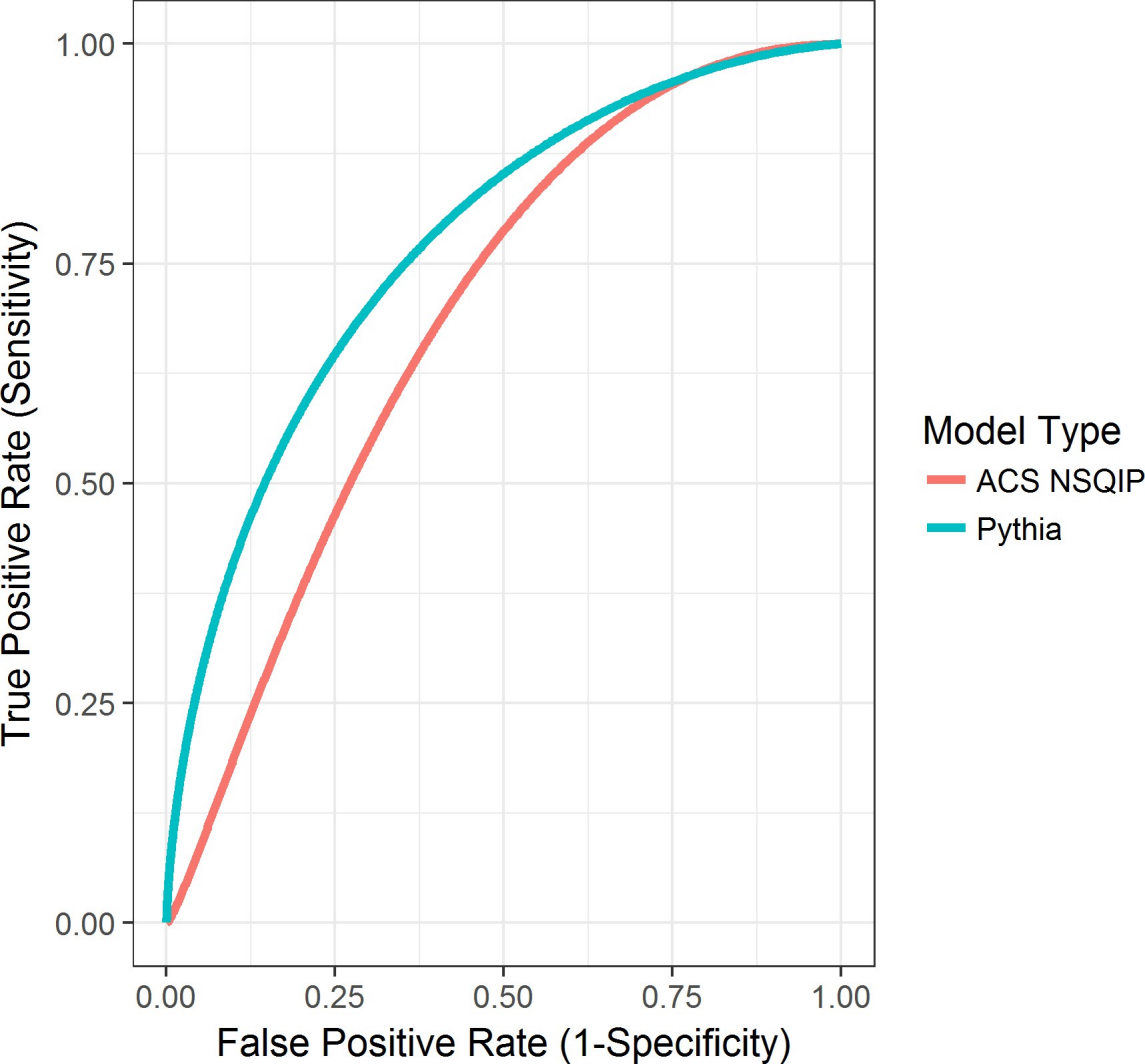
Plotted AUCs for 30-day postoperative mortality in Pythia versus ACS NSQIP. ACS, American College of Surgeons; AUC, area under the receiver operator characteristic curve; NSQIP, National Surgical Quality Improvement Program.

In order to directly compare our model predictions to the ACS NSQIP model predictions, we input preoperative health information from 75 patients in the ACS NSQIP calculator and then in Pythia’s risk calculator. These patients were real patients from our local setting who were randomly selected and were not present within our models’ training set. We upsampled patients with postoperative mortality (16%) to provide more stable estimates of AUC, sensitivity, and specificity. We compared the risk predictions and performance of the 30-day postoperative mortality model and found that Pythia’s model outperformed the NSQIP model by 0.12 AUC (Pythia 0.79 versus NSQIP 0.67) (Fig 5). Furthermore, sensitivity (0.9167 versus 0.7500), specificity (0.5873 versus 0.5556), and PPV (0.2973 versus 0.2432) were also higher for Pythia’s 30-day mortality risk prediction.

## Discussion

We demonstrated that machine learning models built from highly curated, clinically meaningful features from local, structured EHR data were able to achieve high sensitivity and specificity for classifying patients at risk of post-surgical complications. The models and accompanying application can be easily deployed to identify patients for targeted perioperative treatment.

**Fig 6:**
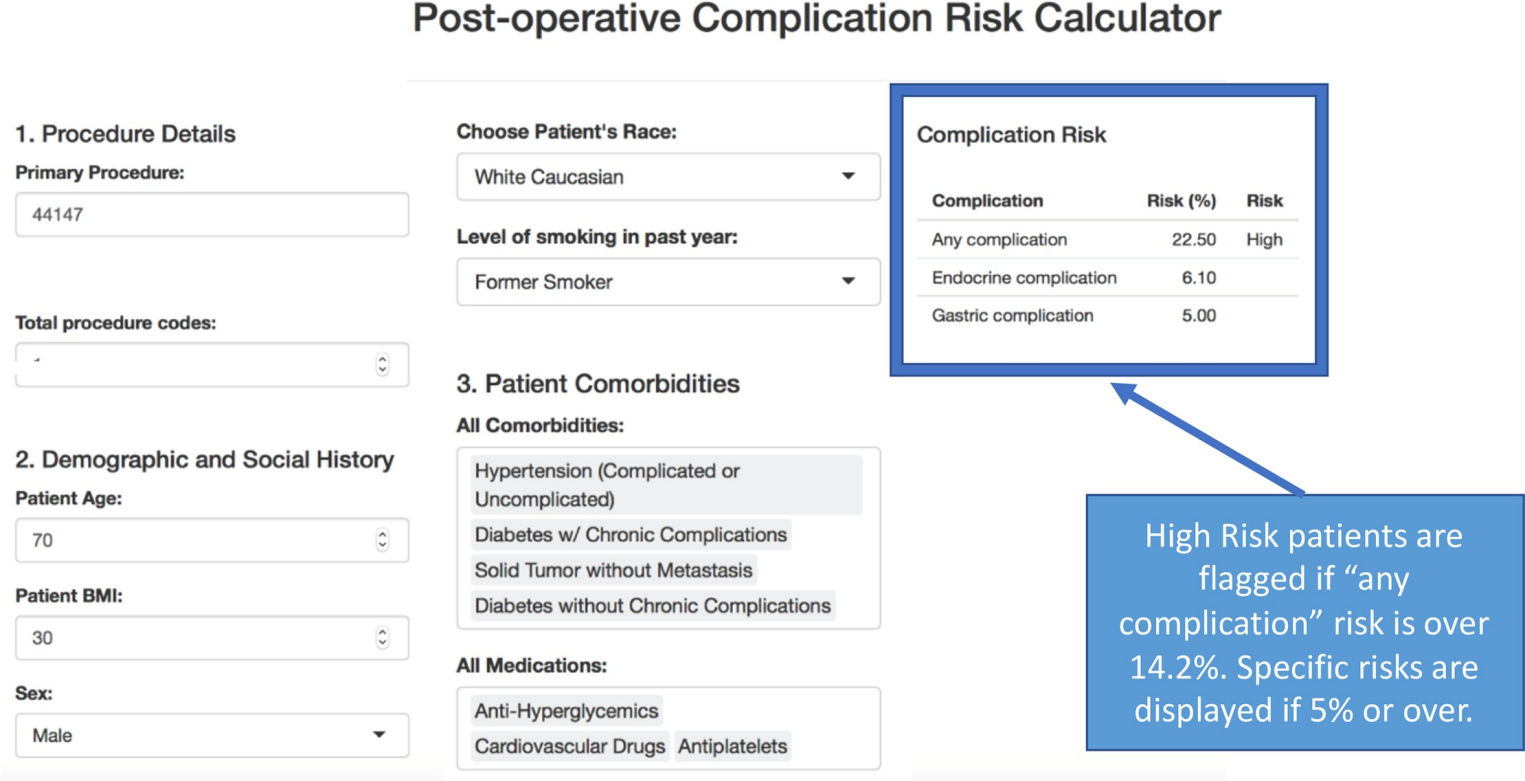
R Shiny application hosting the preoperative risk prediction calculator to use for preoperative patient evaluation in clinic. The calculator includes 9 input fields and calculates a percent risk for each of the 14 postoperative outcomes per patient. The calculator also identifies patients who meet the determined high-risk threshold.

We chose the 14 lasso models to predict complication risk through an online web application built for local clinicians to identify high-risk patients. Our results show that the performances of lasso, random forest, and extreme gradient boosted decision tree models on a non-random, out-of-sample test set from a later time period are nearly identical. However, lasso models performed superiorly to the random forest and extreme gradient boosted decision tree models as a whole, while also returning interpretable coefficients that provide clinicians insights into why patients are at high risk for complications. Lasso models also perform variable selection, minimizing the number of data inputs required in the web application. The variable selection used for the initial pilot of the application, during which manual entry of input features is required, will enable rapid use during clinic visits. The tool requires the input of 9 patient features, curated by grouping the reduced set of covariates chosen by our lasso models, to produce risk scores for 14 postoperative outcome groupings. For example, the comorbidities feature within our calculator is comprised of the 29 binary Elixhauser groupings. This comorbidities feature contains a dropdown menu where multiple comorbidities can be selected if needed. Moreover, Fig 6 demonstrates how the structure of the calculator inputs aligns well with the information collected during surgical clinic visits and with the typical pre-surgical evaluation workflow. As all fields are available as structured data in the EHR system, if the initial pilot is successful, Pythia will enable the rapid deployment of an automated pipeline to extract patient data and calculate risk to notify relevant providers.

Through our sensitivity analysis comparing expert clinical criteria to Pythia’s models, we were able to demonstrate that machine learning models trained from local data can identify individuals at high risk of complications and high cost within the local patient population. Pythia’s models were shown to perform at a higher sensitivity and specificity through this analysis. By specifically targeting a narrower population of patients needing preoperative optimization, our healthcare system can better utilize clinical resources while lowering clinic costs.

Currently, the NSQIP calculator is the most widely used pre-surgical risk prediction model. Recent publications predict postoperative complication risks using ACS NSQIP data [29–33]. These data are manually extracted from EHRs, making them high fidelity but very difficult to update with new patient data or to adjust by adding new variables. Two studies compare models using automatically extracted (EHR) data versus manually collected data. Comparable AUCs were reported by Anderson et al. [34] for multivariate logistic regression models trained on 66 manually collected NSQIP variables versus 25 EHR NSQIP variables. Differences ranged from −0.0073 to 0.1944 across specific surgery procedures for mortality and from 0.0198 to 0.0687 for morbidity [34]. In Amrock et al., the AUCs were 0.813 for mortality and 0.629 for morbidity in multivariate logistic regression models utilizing manually collected data versus 0.795 for mortality and 0.629 for morbidity in the same type of models utilizing EHR data [35]. Both studies found that models using EHR data perform similarly to models using manually extracted data in predicting postoperative morbidity and mortality. However, deploying machine learning models in operations at scale requires automated pipelines for structured EHR data to calculate risk scores and trigger clinical workflows.

The NSQIP calculator uses a logistic regression model using random intercepts per hospital [6], while our models incorporate machine learning via lasso, random forest, and extreme gradient boosted decision trees. We specifically decided not to utilize logistic regression due to the complexity of our patient data. Due to the high collinearity within our dataset and inherent sparsity of many of the covariates, we found that logistic regression suffers from inflated variance of the learned coefficients. Benefits of the lasso model include its ability to perform variable selection, thereby helping to reduce multicollinearity while providing clinical clarity into which predictors cause an increase of specific complication risks. Beyond differences in model choice, the data and validation methodologies differ significantly between the 2 calculators. The ACS NSQIP calculator reports strong predictive in-sample performance, with AUCs of 0.944 for mortality and 0.816 for morbidity [6]. Because the ACS NSQIP models are trained and tested on the same cohort of patients, it is difficult to discern whether these results indicate accurate model predictions or overfitting, limiting the NSQIP calculator’s clinical use capabilities. In comparison, Pythia’s calculator is validated on a non-random, out-of-sample test set from a different time period derived from our health system’s EHRs, with similar AUCs demonstrating strong potential performance in clinical practice with appropriate validation methods.

Our analysis directly comparing the 30-day postoperative mortality models from ACS NSQIP and Pythia demonstrates the superior performance of Pythia’s predictions on our local patients. Many publications have demonstrated the inability of the ACS NSQIP calculator to accurately depict postoperative complication risks in many different patient populations [30–33]. However, very few publications propose superior methods. Not only does this direct comparison between the 2 models provide further evidence that the ACS NSQIP calculator does not perform strongly on our local patients, but it also puts forth a new methodology of local data extraction, curation, and modeling. This new methodology is shown to be superior to ACS NSQIP’s for predicting postoperative complications in a local setting.

Few published postoperative risk prediction models utilizing EHR data exist. SORT (Surgical Outcome Risk Tool) was developed by Protopapa et al. and predicts 30-day mortality utilizing 6 predictor variables using logistic regression, with AUCs ranging from 0.82 to 0.96 for surgical subspecialty groups [36]. To our knowledge, the only other studies utilizing EHR extracted data to build and train machine learning models for postoperative risk predictions are Weller et al. [11] and Soguero-Ruiz et al. [12]. Weller et al. built 5 different types of machine learning models to predict postoperative superficial skin infection, ileus, and bleeding in colorectal surgery cases. However, due to small sample size, their reported AUCs were not as strong, with the exception of random forest models predicting postoperative bleeding complications (AUC 0.8) [11]. Similarly, Soguero-Ruiz et al. used EHR data to predict postoperative anastomosis leakage in colorectal surgeries. Their reported AUC was strong (0.92) using a support vector machine (SVM) model [12]. Our body of work, however, differs greatly from these previously generated postoperative risk prediction models. Not only do we utilize EHR-data-driven machine learning models, but our models have strong predictive performance while predicting postoperative outcomes across a broad range of surgical procedures. In addition, our calculator is based on real-time data extraction from a pipeline from the EHR system that can be continuously and automatically updated and does not rely on manual extraction. The current study addresses a substantial breadth of surgical complications, providing diverse opportunity to intervene on high-risk patients and improve outcomes.

Limitations of our work include missing data, resulting in 99,755 encounters being used to build these models. This number is reduced from the total 145,604 invasive procedure encounters within the data repository. While this is a large reduction in the original data, it still provides a large and sufficient sample to model and predict complications with great accuracy. We chose not to consider imputation methods due to the underlying difficulties of imputing clinical data. Specifically, the most frequent missing variables were outpatient medication lists. The complication groupings within our models are also defined broadly, limiting the user’s ability to understand exactly which type of complication the patient is at risk for within the groupings. For example, cardiac complications include a wide range of ICD-9 and ICD-10 diagnosis codes. Possible missing data also include outpatient death data. The Social Security Death Index excludes a portion of state reported deaths due to public data restrictions as of 2011 [37]. Pythia is a current project that requires optimization with regards to missing data and further curation of fields from additional tables in the EHR database. Efforts to develop strategies for effective imputation, data curation, and the addition of other quality data sources are priorities for future iterations.

Further analytical limitations include our 30-day mortality model comparison to ACS NSQIP’s model performance. This analysis was based on a random sample of 75 patients. Analyses with larger patient sample sizes will be needed in the future.

Although our proposed data collection methodology is less burdensome due to non-manual data extraction reducing cost and time, as with all data collection methods, there are limitations. Over time, standards of data collection within the EHR system may change as well as clinical practice trends, both altering the way data are represented in large EHR data repositories. Monitoring systems that are able to catch these variations need to be put in place as these healthcare repositories continue to grow over long periods of time. Efforts to develop the monitoring architecture around EHR repositories are also priorities for future iterations of this surgical data repository.

Our work demonstrates that we can better identify patients who are high risk and high cost within our referral base by creating a site-specific surgical data pipeline and repository to fuel our clinical calculator. Our calculator is unique and personalized to our institution because it is derived from our local patient population, with our university-affiliated surgeons. As stated by Bates et al., “algorithms are most effective and perform best when they are derived from and then used in similar populations” [38], thus further highlighting the need for local data to drive healthcare insights. By leveraging our local institution’s EHR data, not only are we able to easily build machine learning models to improve healthcare delivery to our patients, but we also have the ability to enhance our education for trainees and build future quality improvement initiatives. In addition, our calculator is in the form of a clinical portal on a web application for easy usability. By quickly inputting a surgical patient’s information into the 9 fields of the calculator, a clinician can see if the patient is deemed high risk, thereby requiring further preoperative evaluation and prompting referral to a high-risk clinic. Displays of the risk attributable to a given disease or medication can also help the team prioritize preoperative interventions and postoperative monitoring and care that have been shown to significantly lower postoperative complications as well as length of stay [5]. Use of the tool may also promote more specific discussions about the benefits and risks of surgery with patients, enhance shared decision-making, and advance care planning. If implemented thoughtfully within a preoperative clinic workflow, this tool has the ability to help support decisions made by a patient’s care team. Hosted through a simple web application, our risk calculator can be easily incorporated into the EHR system and can be automatically populated as patient features are being input into the chart. Plans to integrate this calculator into our institute’s EHR system are currently underway. Once fully implemented, the models would be updated on a yearly basis by retraining and validating them with the latest patient data. Through this yearly retrain and validation plan, we will be able to track any changes made to our EHR data collection system and determine whether our models are performing strongly over time. Furthermore, as a project within our institute’s learning health system, we plan to reevaluate our implementation as a whole and make correctional adjustments on a continual basis to best support our providers in their decision-making process. We believe that our methods for building a data pipeline from EHRs in order to develop machine learning models create a prototype for an institutional learning health system. In the future, our methods can be disseminated to develop infrastructure and best practices to extend to other institutions and patient populations in order to improve patient care at other healthcare institutions.

## Supporting information

S1 Checklist TRIPOD checklist. (DOCX)

S1 Table Data variables used in the 3 machine learning methodologies. (DOCX)

S2 Table Flagged ICD codes to identify complications. (DOCX)

## Abbreviations

ACS: American College of Surgeons
AUC: area under the receiver operator characteristic curve
CPT: Current Procedural Terminology
DUHS: Duke University Health System
EHR: electronic health record
ICD: International Classification of Diseases
lasso: least absolute shrinkage and selection operator
NSQIP: National Surgical Quality Improvement Program
PPV: positive predictive value
