# Supplementary material for: Development and validation of machine learning models to identify high-risk surgical patients using automatically curated electronic health record data (Pythia): A retrospective, single-site study
Source: PLoS Med. 2018 Nov 27;15(11):e1002701. doi: 10.1371/journal.pmed.1002701 (PMC6258507; doi:10.1371/journal.pmed.1002701)
Supplement: S1 Table — (DOCX) [file pmed.1002701.s002.docx]

**S1 Table: Data variables used in three machine learning methodologies**

**Table 1.** All patient data variables used to train and test machine learning models

| Variable Type (n) | Variables |
| --- | --- |
| Demographics (3) | Age, Gender, Race, Smoking Status |
| Other Encounter Information | Admission Date, Discharge Date, Surgery Specialty |
| Elixhauser Comorbidities Groupings (29) | Congestive Heart Failure, Fluid and Electrolyte Disorders, Paralysis, Valvular Disease, Pulmonary Circulation Disorders, Deficiency Anemias, Other Neurological, Disorders ,Blood Loss Anemia, Hypertension (Complicated or Uncomplicated), Renal Failure ,Weight Loss ,Coagulation Deficiency,  Chronic Pulmonary Disease, Psychoses, Diabetes without Chronic Complications, Diabetes with Chronic Complications, Metastatic Cancer, Peptic Ulcer Disease, Rheumatoid Arthritis, Hypothyroidism, Liver Disease, Depression, Solid Tumor without Metastasis, Drug Abuse, Lymphoma, HIV or AIDS, Peripheral Vascular Disease, Alcohol Abuse, Obesity |
| Therapeutic Medication Classes (15) | Anticoagulants, Anesthetics, Antibiotics, Cardiac Medications , Analgesics, Anti-Hyperglycemics, Anti-Neoplastics, Antiplatelets, Antivirals, Autonomic Drugs, Cardiovascular Drugs , CNS Drugs, Diuretics, Hormones, Immunosuppressant |
| Complication Groupings (14) | Any Complication, Cardiac Complication, Endocrine Complication, Gastric Complication, Genitourinary Complication, Hematologic Complication, Integumentary Complication, Neurological Complication, Pulmonary Complication, Renal Complication, Sepsis Complication, Shock Complication, Vascular Complication, 90-day Mortality |
| CCS Classes (128) | Incision and excision of CNS, Thyroidectomy partial or complete, Endoscopy and endoscopic biopsy of the urinary tract, Transurethral excision/drainage/removal urinary obstruction, Ureteral catheterization, Nephrotomy and nephrostomy, Nephrectomy partial or complete, Kidney transplant, Genitourinary incontinence procedures, Procedures on the urethra, Other OR therapeutic procedures of urinary tract, Transurethral resection of prostate (TURP), Open prostatectomy, Other non-OR therapeutic procedures, male genital, Other OR therapeutic procedures, male genital, Oophorectomy, unilateral and bilateral, Other therapeutic endocrine procedures, Other operations on ovary, Ligation of fallopian tubes, Removal of ectopic pregnancy, Other operations on fallopian tubes, Hysterectomy, abdominal and vaginal, Other excision of cervix and uterus, Abortion (termination of pregnancy), Repair of cystocele and rectocele, obliteration of vaginal vault, Corneal transplant, Other diagnostic procedures, female organs, Other OR therapeutic procedures, female organs, Cesarean section, Glaucoma procedures, Other therapeutic obstetrical procedures, Partial excision bone, Bunionectomy or repair of toe deformities, Treatment, facial fracture or dislocation, Treatment, fracture or dislocation of radius and ulna, Treatment, fracture or dislocation of hip and femur  Treatment, fracture or dislocation of lower extremity (other than hip or femur), Other fracture and dislocation procedure, Arthroscopy, Lens and cataract procedures,  Division of joint capsule, ligament or cartilage, Excision of semilunar cartilage of knee, Arthroplasty knee, Hip replacement, total and partial, Arthroplasty other than hip or knee, Amputation of lower extremity, Spinal fusion, Other diagnostic procedures on musculoskeletal system, Repair of retinal tear, detachment, Other therapeutic procedures on muscles and tendons, Other OR therapeutic procedures on bone, Other OR therapeutic procedures on joints, Other non-OR therapeutic procedures on musculoskeletal system, Other OR therapeutic procedures on musculoskeletal system, Lumpectomy, quadrantectomy of breast, Mastectomy, Incision and drainage, skin and subcutaneous tissue, Debridement of wound, infection or burn, Destruction of lesion of retina and choroid, Excision of skin lesion, Suture of skin and subcutaneous tissue, Skin graft, Other non-OR therapeutic procedures on skin and breast, Other OR therapeutic procedures on skin and breast, Other organ transplantation, Other therapeutic procedures on eyelids, conjunctiva, cornea, Insertion, replacement, or removal of extracranial ventricular shunt, Other intraocular therapeutic procedures, Other extraocular muscle and orbit therapeutic procedures, Traction, splints, and other wound care, Tympanoplasty, Nonoperative removal of foreign body, Myringotomy, Mastoidectomy, Gastric bypass and volume reduction, Other therapeutic ear procedures, Control of epistaxis, Plastic procedures on nose, Oral and Dental Services, Laminectomy, excision intervertebral disc, Tonsillectomy and/or adenoidectomy, Other OR therapeutic procedures on nose, mouth and pharynx, Tracheostomy, temporary and permanent, Lobectomy or pneumonectomy, Other diagnostic procedures on lung and bronchus  Incision of pleura, thoracentesis, chest drainage, Other OR therapeutic procedures on respiratory system, Heart valve procedures, Coronary artery bypass graft (CABG), Insertion, revision, replacement, removal of cardiac pacemaker or cardioverter/defibrillator, Other OR heart procedures, Extracorporeal circulation auxiliary to open heart procedures, Endarterectomy, vessel of head and neck, Aortic resection, replacement or anastomosis, Varicose vein stripping, lower limb, Other vascular catheterization, not heart, Peripheral vascular bypass, Other vascular bypass and shunt, not heart, Creation, revision and removal of arteriovenous fistula or vessel-to-vessel cannula for dialysis, Other OR procedures on vessels of head and neck, Decompression peripheral nerve, Embolectomy and endarterectomy of lower limbs, Other OR procedures on vessels other than head and neck, Other non-OR therapeutic cardiovascular procedures, Procedures on spleen, Other therapeutic procedures, hemic and lymphatic system, Other diagnostic nervous system procedures, Gastrostomy, temporary and permanent, Colostomy, temporary and permanent, Ileostomy and other enterostomy, Gastrectomy, partial and total, Small bowel resection, Colonoscopy and biopsy, Colorectal resection, Local excision of large intestine lesion (not endoscopic), Appendectomy, Cholecystectomy and common duct exploration, Inguinal and femoral hernia repair, Other hernia repair, Laparoscopy, Exploratory laparotomy, Other OR therapeutic nervous system procedures  Excision, lysis peritoneal adhesions, Other bowel diagnostic procedures, Other OR upper GI therapeutic procedures, Other non-OR lower GI therapeutic procedures, Other OR lower GI therapeutic procedures, Other OR gastrointestinal therapeutic procedures |
