# Supplementary material for: Development and validation of machine learning models to identify high-risk surgical patients using automatically curated electronic health record data (Pythia): A retrospective, single-site study
Source: PLoS Med. 2018 Nov 27;15(11):e1002701. doi: 10.1371/journal.pmed.1002701 (PMC6258507; doi:10.1371/journal.pmed.1002701)
Supplement: S2 Table — (DOCX) [file pmed.1002701.s003.docx]

**S2 Table:** Flagged ICD codes to identify complications

**Table 2.** 271 flagged ICD codes input within 30 days following a procedure data to identify postoperative complications.

| 410.00 | Acute myocardial infarction of anterolateral wall, episode of care unspecified |
| --- | --- |
| 410.01 | Acute myocardial infarction of anterolateral wall, initial episode of care |
| 410.02 | Acute myocardial infarction of anterolateral wall, subsequent episode of care |
| 427.5 | Cardiac arrest |
| 427.0 | Paroxysmal supraventricular tachycardia |
| 428.0 | Congestive heart failure, unspecified |
| 410.10 | Acute myocardial infarction of other anterior wall, episode of care unspecified |
| 410.11 | Acute myocardial infarction of other anterior wall, initial episode of care |
| 410.12 | Acute myocardial infarction of other anterior wall, subsequent episode of care |
| 427.41 | Ventricular fibrillation |
| 427.42 | Ventricular flutter |
| 427.1 | Paroxysmal ventricular tachycardia |
| 428.21 | Acute systolic heart failure |
| 410.20 | Acute myocardial infarction of inferolateral wall, episode of care unspecified |
| 410.21 | Acute myocardial infarction of inferolateral wall, initial episode of care |
| 410.22 | Acute myocardial infarction of inferolateral wall, subsequent episode of care |
| 427.41 | Ventricular fibrillation |
| 427.2 | Paroxysmal tachycardia, unspecified |
| 428.23 | Acute on chronic systolic heart failure |
| 410.30 | Acute myocardial infarction of inferoposterior wall, episode of care unspecified |
| 410.31 | Acute myocardial infarction of inferoposterior wall, initial episode of care |
| 410.32 | Acute myocardial infarction of inferoposterior wall, subsequent episode of care |
| 427.42 | Ventricular flutter |
| 427.31 | Atrial fibrillation |
| 427.32 | Atrial flutter |
| 428.31 | Acute diastolic heart failure |
| 410.40 | Acute myocardial infarction of other inferior wall, episode of care unspecified |
| 410.41 | Acute myocardial infarction of other inferior wall, initial episode of care |
| 410.42 | Acute myocardial infarction of other inferior wall, subsequent episode of care |
| 997.1 | Cardiac complications, not elsewhere classified |
| 427.31 | Atrial fibrillation |
| 428.33 | Acute on chronic diastolic heart failure |
| 410.50 | Acute myocardial infarction of other lateral wall, episode of care unspecified |
| 410.51 | Acute myocardial infarction of other lateral wall, initial episode of care |
| 410.52 | Acute myocardial infarction of other lateral wall, subsequent episode of care |
| 427.5 | Cardiac arrest |
| 427.32 | Atrial flutter |
| 428.41 | Acute combined systolic and diastolic heart failure |
| 410.60 | True posterior wall infarction, episode of care unspecified |
| 410.61 | True posterior wall infarction, initial episode of care |
| 410.62 | True posterior wall infarction, subsequent episode of care |
| 997.1 | Cardiac complications, not elsewhere classified |
| 427.60 | Premature beats, unspecified |
| 428.43 | Acute on chronic combined systolic and diastolic heart failure |
| 410.70 | Subendocardial infarction, episode of care unspecified |
| 410.71 | Subendocardial infarction, initial episode of care |
| 410.72 | Subendocardial infarction, subsequent episode of care |
| 427.81 | Sinoatrial node dysfunction |
| 427.89 | Other specified cardiac dysrhythmias |
| 428.0 | Congestive heart failure, unspecified |
| 428.1 | Left heart failure |
| 428.20 | Systolic heart failure, unspecified |
| 428.21 | Acute systolic heart failure |
| 428.22 | Chronic systolic heart failure |
| 428.23 | Acute on chronic systolic heart failure |
| 428.30 | Diastolic heart failure, unspecified |
| 428.31 | Acute diastolic heart failure |
| 428.32 | Chronic diastolic heart failure |
| 428.33 | Acute on chronic diastolic heart failure |
| 428.40 | Combined systolic and diastolic heart failure, unspecified |
| 428.41 | Acute combined systolic and diastolic heart failure |
| 428.42 | Chronic combined systolic and diastolic heart failure |
| 428.43 | Acute on chronic combined systolic and diastolic heart failure |
| 428.9 | Heart failure, unspecified |
| 410.80 | Acute myocardial infarction of other specified sites, episode of care unspecified |
| 410.81 | Acute myocardial infarction of other specified sites, initial episode of care |
| 410.82 | Acute myocardial infarction of other specified sites, subsequent episode of care |
| 427.89 | Other specified cardiac dysrhythmias |
| 428.1 | Left heart failure |
| 410.90 | Acute myocardial infarction of unspecified site, episode of care unspecified |
| 410.91 | Acute myocardial infarction of unspecified site, initial episode of care |
| 410.92 | Acute myocardial infarction of unspecified site, subsequent episode of care |
| 427.0 | Paroxysmal supraventricular tachycardia |
| 427.1 | Paroxysmal ventricular tachycardia |
| 427.2 | Paroxysmal tachycardia, unspecified |
| 427.31 | Atrial fibrillation |
| 427.32 | Atrial flutter |
| 427.41 | Ventricular fibrillation |
| 427.42 | Ventricular flutter |
| 427.5 | Cardiac arrest |
| 427.60 | Premature beats, unspecified |
| 427.61 | Supraventricular premature beats |
| 427.69 | Other premature beats |
| 427.81 | Sinoatrial node dysfunction |
| 427.89 | Other specified cardiac dysrhythmias |
| 427.9 | Cardiac dysrhythmia, unspecified |
| 410.00 | Acute myocardial infarction of anterolateral wall, episode of care unspecified |
| 410.01 | Acute myocardial infarction of anterolateral wall, initial episode of care |
| 410.02 | Acute myocardial infarction of anterolateral wall, subsequent episode of care |
| 410.10 | Acute myocardial infarction of other anterior wall, episode of care unspecified |
| 410.11 | Acute myocardial infarction of other anterior wall, initial episode of care |
| 410.12 | Acute myocardial infarction of other anterior wall, subsequent episode of care |
| 410.20 | Acute myocardial infarction of inferolateral wall, episode of care unspecified |
| 410.21 | Acute myocardial infarction of inferolateral wall, initial episode of care |
| 410.22 | Acute myocardial infarction of inferolateral wall, subsequent episode of care |
| 410.30 | Acute myocardial infarction of inferoposterior wall, episode of care unspecified |
| 410.31 | Acute myocardial infarction of inferoposterior wall, initial episode of care |
| 410.32 | Acute myocardial infarction of inferoposterior wall, subsequent episode of care |
| 410.40 | Acute myocardial infarction of other inferior wall, episode of care unspecified |
| 410.41 | Acute myocardial infarction of other inferior wall, initial episode of care |
| 410.42 | Acute myocardial infarction of other inferior wall, subsequent episode of care |
| 410.50 | Acute myocardial infarction of other lateral wall, episode of care unspecified |
| 410.51 | Acute myocardial infarction of other lateral wall, initial episode of care |
| 410.52 | Acute myocardial infarction of other lateral wall, subsequent episode of care |
| 410.60 | True posterior wall infarction, episode of care unspecified |
| 410.61 | True posterior wall infarction, initial episode of care |
| 410.62 | True posterior wall infarction, subsequent episode of care |
| 410.70 | Subendocardial infarction, episode of care unspecified |
| 410.71 | Subendocardial infarction, initial episode of care |
| 410.72 | Subendocardial infarction, subsequent episode of care |
| 410.80 | Acute myocardial infarction of other specified sites, episode of care unspecified |
| 410.81 | Acute myocardial infarction of other specified sites, initial episode of care |
| 410.82 | Acute myocardial infarction of other specified sites, subsequent episode of care |
| 410.90 | Acute myocardial infarction of unspecified site, episode of care unspecified |
| 410.91 | Acute myocardial infarction of unspecified site, initial episode of care |
| 410.92 | Acute myocardial infarction of unspecified site, subsequent episode of care |
| 427.0 | Paroxysmal supraventricular tachycardia |
| 410.00 | Acute myocardial infarction of anterolateral wall, episode of care unspecified |
| 291.0 | Alcohol withdrawal delirium |
| 291.1 | Alcohol-induced persisting amnestic disorder |
| 291.2 | Alcohol-induced persisting dementia |
| 291.3 | Alcohol-induced psychotic disorder with hallucinations |
| 291.4 | Idiosyncratic alcohol intoxication |
| 291.5 | Alcohol-induced psychotic disorder with delusions |
| 291.81 | Alcohol withdrawal |
| 291.82 | Alcohol induced sleep disorders |
| 291.89 | Other alcohol-induced mental disorders |
| 291.9 | Unspecified alcohol-induced mental disorders |
| 251.2 | Hypoglycemia, unspecified |
| 790.29 | Other abnormal glucose |
| 251.2 | Hypoglycemia, unspecified |
| E884.2 | Accidental fall from chair |
| E884.3 | Accidental fall from wheelchair |
| E884.4 | Accidental fall from bed |
| E884.5 | Accidental fall from other furniture |
| E884.6 | Accidental fall from commode |
| E884.9 | Other accidental fall from one level to another |
| E885.9 | Fall from other slipping, tripping, or stumbling |
| E886.9 | Other and unspecified falls on same level from collision, pushing, or shoving, by or with other person |
| E888.0 | Fall resulting in striking against sharp object |
| E888.1 | Fall resulting in striking against other object |
| E888.8 | Other fall |
| E888.9 | Unspecified fall |
| 787.01 | Nausea with vomiting |
| 787.02 | Nausea alone |
| 787.03 | Vomiting alone |
| 787.04 | Bilious emesis |
| 560.1 | Paralytic ileus |
| 787.01 | Nausea with vomiting |
| 537.2 | Chronic duodenal ileus |
| 787.02 | Nausea alone |
| 560.31 | Gallstone ileus |
| 787.03 | Vomiting alone |
| 787.04 | Bilious emesis |
| 787.01 | Nausea with vomiting |
| 787.02 | Nausea alone |
| 787.03 | Vomiting alone |
| 787.04 | Bilious emesis |
| 599.0 | Urinary tract infection, site not specified |
| 788.20 | Retention of urine, unspecified |
| 599.0 | Urinary tract infection, site not specified |
| 599.1 | Urethral fistula |
| 599.2 | Urethral diverticulum |
| 599.3 | Urethral caruncle |
| 599.4 | Urethral false passage |
| 599.5 | Prolapsed urethral mucosa |
| 599.60 | Urinary obstruction, unspecified |
| 599.69 | Urinary obstruction, not elsewhere classified |
| 599.70 | Hematuria, unspecified |
| 599.71 | Gross hematuria |
| 599.72 | Microscopic hematuria |
| 599.81 | Urethral hypermobility |
| 599.82 | Intrinsic (urethral) sphincter deficiency [ISD] |
| 599.83 | Urethral instability |
| 599.84 | Other specified disorders of urethra |
| 599.89 | Other specified disorders of urinary tract |
| 599.9 | Unspecified disorder of urethra and urinary tract |
| 788.21 | Incomplete bladder emptying |
| 788.20 | Retention of urine, unspecified |
| 788.21 | Incomplete bladder emptying |
| 788.29 | Other specified retention of urine |
| 285.1 | Acute posthemorrhagic anemia |
| 287.9 | Unspecified hemorrhagic conditions |
| 998.11 | Hemorrhage complicating a procedure |
| 998.12 | Hematoma complicating a procedure |
| 998.13 | Seroma complicating a procedure |
| 998.11 | Hemorrhage complicating a procedure |
| 998.12 | Hematoma complicating a procedure |
| E870.0 | Accidental cut, puncture, perforation or hemorrhage during surgical operation |
| 729.92 | Nontraumatic hematoma of soft tissue |
| 285.1 | Acute posthemorrhagic anemia |
| 287.9 | Unspecified hemorrhagic conditions |
| 707.20 | Pressure ulcer, unspecified stage |
| 998.30 | Disruption of wound, unspecified |
| 998.31 | Disruption of internal operation (surgical) wound |
| 998.32 | Disruption of external operation (surgical) wound |
| 998.33 | Disruption of traumatic injury wound repair |
| 998.51 | Infected postoperative seroma |
| 998.59 | Other postoperative infection |
| 707.21 | Pressure ulcer, stage I |
| 998.30 | Disruption of wound, unspecified |
| 998.51 | Infected postoperative seroma |
| 998.59 | Other postoperative infection |
| 707.22 | Pressure ulcer, stage II |
| 998.31 | Disruption of internal operation (surgical) wound |
| 998.51 | Infected postoperative seroma |
| 707.23 | Pressure ulcer, stage III |
| 998.32 | Disruption of external operation (surgical) wound |
| 998.59 | Other postoperative infection |
| 707.24 | Pressure ulcer, stage IV |
| 998.83 | Non-healing surgical wound |
| 707.25 | Pressure ulcer, unstageable |
| 780.09 | Other alteration of consciousness |
| 290.11 | Presenile dementia with delirium |
| 434.01 | Cerebral thrombosis with cerebral infarction |
| 780.01 | Coma |
| 293.0 | Delirium due to conditions classified elsewhere |
| 290.3 | Senile dementia with delirium |
| 434.11 | Cerebral embolism with cerebral infarction |
| 780.03 | Persistent vegetative state |
| 780.97 | Altered mental status |
| 290.41 | Vascular dementia, with delirium |
| 434.91 | Cerebral artery occlusion, unspecified with cerebral infarction |
| 780.09 | Other alteration of consciousness |
| 292.81 | Drug-induced delirium |
| 291.0 | Alcohol withdrawal delirium |
| 291.0 | Alcohol withdrawal delirium |
| 997.00 | Nervous system complication, unspecified |
| 997.01 | Central nervous system complication |
| 997.02 | Iatrogenic cerebrovascular infarction or hemorrhage |
| 997.09 | Other nervous system complications |
| 348.30 | Encephalopathy, unspecified |
| 348.31 | Metabolic encephalopathy |
| 348.39 | Other encephalopathy |
| 292.81 | Drug-induced delirium |
| 997.02 | Iatrogenic cerebrovascular infarction or hemorrhage |
| 997.00 | Nervous system complication, unspecified |
| 348.39 | Other encephalopathy |
| 293.0 | Delirium due to conditions classified elsewhere |
| 436 | Acute, but ill-defined, cerebrovascular disease |
| 997.01 | Central nervous system complication |
| 293.1 | Subacute delirium |
| 997.09 | Other nervous system complications |
| 780.09 | Other alteration of consciousness |
| 348.30 | Encephalopathy, unspecified |
| 348.31 | Metabolic encephalopathy |
| 348.39 | Other encephalopathy |
| 349.82 | Toxic encephalopathy |
| 437.2 | Hypertensive encephalopathy |
| 572.2 | Hepatic encephalopathy |
| 768.70 | Hypoxic-ischemic encephalopathy, unspecified |
| 768.71 | Mild hypoxic-ischemic encephalopathy |
| 768.72 | Moderate hypoxic-ischemic encephalopathy |
| 768.73 | Severe hypoxic-ischemic encephalopathy |
| 768.71 | Mild hypoxic-ischemic encephalopathy |
| 768.72 | Moderate hypoxic-ischemic encephalopathy |
| 768.73 | Severe hypoxic-ischemic encephalopathy |
| 780.97 | Altered mental status |
| 298.2 | Reactive confusion |
| 518.0 | Pulmonary collapse |
| 518.1 | Interstitial emphysema |
| 518.2 | Compensatory emphysema |
| 518.3 | Pulmonary eosinophilia |
| 518.4 | Acute edema of lung, unspecified |
| 518.51 | Acute respiratory failure following trauma and surgery |
| 518.52 | Other pulmonary insufficiency, not elsewhere classified, following trauma and surgery |
| 518.53 | Acute and chronic respiratory failure following trauma and surgery |
| 518.6 | Allergic bronchopulmonary aspergillosis |
| 518.7 | Transfusion related acute lung injury (TRALI) |
| 518.81 | Acute respiratory failure |
| 518.82 | Other pulmonary insufficiency, not elsewhere classified |
| 518.83 | Chronic respiratory failure |
| 518.84 | Acute and chronic respiratory failure |
| 518.89 | Other diseases of lung, not elsewhere classified |
| 507.0 | Pneumonitis due to inhalation of food or vomitus |
| V46.13 | Encounter for weaning from respirator [ventilator] |
| V46.11 | Dependence on respirator, status |
| 518.51 | Acute respiratory failure following trauma and surgery |
| 518.52 | Other pulmonary insufficiency, not elsewhere classified, following trauma and surgery |
| 518.53 | Acute and chronic respiratory failure following trauma and surgery |
| 112.4 | Candidiasis of lung |
| 518.51 | Acute respiratory failure following trauma and surgery |
| 518.51 | Acute respiratory failure following trauma and surgery |
| 518.52 | Other pulmonary insufficiency, not elsewhere classified, following trauma and surgery |
| 482.0 | Pneumonia due to Klebsiella pneumoniae |
| 518.53 | Acute and chronic respiratory failure following trauma and surgery |
| 482.1 | Pneumonia due to Pseudomonas |
| 518.81 | Acute respiratory failure |
| 482.30 | Pneumonia due to Streptococcus, unspecified |
| 518.82 | Other pulmonary insufficiency, not elsewhere classified |
| 482.31 | Pneumonia due to Streptococcus, group A |
| 518.84 | Acute and chronic respiratory failure |
| 482.32 | Pneumonia due to Streptococcus, group B |
| 799.1 | Respiratory arrest |
| 482.39 | Pneumonia due to other Streptococcus |
| V46.11 | Dependence on respirator, status |
| 482.40 | Pneumonia due to Staphylococcus, unspecified |
| V46.2 | Other dependence on machines, supplemental oxygen |
| 482.41 | Methicillin susceptible pneumonia due to Staphylococcus aureus |
| 482.42 | Methicillin resistant pneumonia due to Staphylococcus aureus |
| 482.49 | Other Staphylococcus pneumonia |
| 482.81 | Pneumonia due to anaerobes |
| 482.82 | Pneumonia due to escherichia coli [E. coli] |
| 482.83 | Pneumonia due to other gram-negative bacteria |
| 482.89 | Pneumonia due to other specified bacteria |
| 482.9 | Bacterial pneumonia, unspecified |
| 483.8 | Pneumonia due to other specified organism |
| 484.8 | Pneumonia in other infectious diseases classified elsewhere |
| 484.8 | Pneumonia in other infectious diseases classified elsewhere |
| 484.8 | Pneumonia in other infectious diseases classified elsewhere |
| 513.0 | Abscess of lung |
| 507.0 | Pneumonitis due to inhalation of food or vomitus |
| 507.1 | Pneumonitis due to inhalation of oils and essences |
| 507.8 | Pneumonitis due to other solids and liquids |
| 481 | Pneumococcal pneumonia [Streptococcus pneumoniae pneumonia] |
| 482.0 | Pneumonia due to Klebsiella pneumoniae |
| 482.1 | Pneumonia due to Pseudomonas |
| 482.2 | Pneumonia due to Hemophilus influenzae [H. influenzae] |
| 482.30 | Pneumonia due to Streptococcus, unspecified |
| 482.31 | Pneumonia due to Streptococcus, group A |
| 482.32 | Pneumonia due to Streptococcus, group B |
| 482.39 | Pneumonia due to other Streptococcus |
| 482.40 | Pneumonia due to Staphylococcus, unspecified |
| 482.41 | Methicillin susceptible pneumonia due to Staphylococcus aureus |
| 482.42 | Methicillin resistant pneumonia due to Staphylococcus aureus |
| 482.49 | Other Staphylococcus pneumonia |
| 482.81 | Pneumonia due to anaerobes |
| 482.82 | Pneumonia due to escherichia coli [E. coli] |
| 482.83 | Pneumonia due to other gram-negative bacteria |
| 482.84 | Pneumonia due to Legionnaires' disease |
| 482.89 | Pneumonia due to other specified bacteria |
| 482.9 | Bacterial pneumonia, unspecified |
| 483.0 | Pneumonia due to mycoplasma pneumoniae |
| 483.1 | Pneumonia due to chlamydia |
| 483.8 | Pneumonia due to other specified organism |
| 484.1 | Pneumonia in cytomegalic inclusion disease |
| 484.3 | Pneumonia in whooping cough |
| 484.5 | Pneumonia in anthrax |
| 484.6 | Pneumonia in aspergillosis |
| 484.7 | Pneumonia in other systemic mycoses |
| 484.8 | Pneumonia in other infectious diseases classified elsewhere |
| 485 | Bronchopneumonia, organism unspecified |
| 486 | Pneumonia, organism unspecified |
| 513.0 | Abscess of lung |
| 584.5 | Acute kidney failure with lesion of tubular necrosis |
| 584.6 | Acute kidney failure with lesion of renal cortical necrosis |
| 584.9 | Acute kidney failure, unspecified |
| 586 | Renal failure, unspecified |
| 593.0 | Nephroptosis |
| 586 | Renal failure, unspecified |
| 583.9 | Nephritis and nephropathy, not specified as acute or chronic, with unspecified pathological lesion in kidney |
| 999.32 | Bloodstream infection due to central venous catheter |
| 995.91 | Sepsis |
| 995.90 | Systemic inflammatory response syndrome, unspecified |
| 995.91 | Sepsis |
| 995.92 | Severe sepsis |
| 995.93 | Systemic inflammatory response syndrome due to noninfectious process without acute organ dysfunction |
| 995.94 | Systemic inflammatory response syndrome due to noninfectious process with acute organ dysfunction |
| 909.3 | Late effect of complications of surgical and medical care |
| 785.52 | Septic shock |
| 038.0 | Streptococcal septicemia |
| 038.10 | Staphylococcal septicemia, unspecified |
| 038.11 | Methicillin susceptible Staphylococcus aureus septicemia |
| 038.12 | Methicillin resistant Staphylococcus aureus septicemia |
| 038.19 | Other staphylococcal septicemia |
| 038.2 | Pneumococcal septicemia [Streptococcus pneumoniae septicemia] |
| 038.3 | Septicemia due to anaerobes |
| 038.40 | Septicemia due to gram-negative organism, unspecified |
| 038.41 | Septicemia due to hemophilus influenzae [H. influenzae] |
| 038.42 | Septicemia due to escherichia coli [E. coli] |
| 038.43 | Septicemia due to pseudomonas |
| 038.44 | Septicemia due to serratia |
| 038.49 | Other septicemia due to gram-negative organisms |
| 038.8 | Other specified septicemias |
| 038.9 | Unspecified septicemia |
| 038.9 | Unspecified septicemia |
| 995.92 | Severe sepsis |
| 998.02 | Postoperative shock, septic |
| 970.0 | Poisoning by analeptics |
| 970.1 | Poisoning by opiate antagonists |
| 970.81 | Poisoning by cocaine |
| 970.89 | Poisoning by other central nervous system stimulants |
| 970.9 | Poisoning by unspecified central nervous system stimulant |
| 458.29 | Other iatrogenic hypotension |
| 998.00 | Postoperative shock, unspecified |
| 998.01 | Postoperative shock, cardiogenic |
| 998.02 | Postoperative shock, septic |
| 998.09 | Postoperative shock, other |
| 998.01 | Postoperative shock, cardiogenic |
| 998.09 | Postoperative shock, other |
| 785.51 | Cardiogenic shock |
| 453.40 | Acute venous embolism and thrombosis of unspecified deep vessels of lower extremity |
| 453.41 | Acute venous embolism and thrombosis of deep vessels of proximal lower extremity |
| 453.42 | Acute venous embolism and thrombosis of deep vessels of distal lower extremity |
| 415.19 | Other pulmonary embolism and infarction |
| 453.81 | Acute venous embolism and thrombosis of superficial veins of upper extremity |
| 453.82 | Acute venous embolism and thrombosis of deep veins of upper extremity |
| 453.83 | Acute venous embolism and thrombosis of upper extremity, unspecified |
| 453.84 | Acute venous embolism and thrombosis of axillary veins |
| 453.85 | Acute venous embolism and thrombosis of subclavian veins |
| 453.86 | Acute venous embolism and thrombosis of internal jugular veins |
| 453.87 | Acute venous embolism and thrombosis of other thoracic veins |
| 453.89 | Acute venous embolism and thrombosis of other specified veins |
| 415.12 | Septic pulmonary embolism |
| 453.9 | Other venous embolism and thrombosis of unspecified site |
| 415.13 | Saddle embolus of pulmonary artery |
| 453.41 | Acute venous embolism and thrombosis of deep vessels of proximal lower extremity |
| 415.11 | Iatrogenic pulmonary embolism and infarction |
| 453.42 | Acute venous embolism and thrombosis of deep vessels of distal lower extremity |
| 453.40 | Acute venous embolism and thrombosis of unspecified deep vessels of lower extremity |
| 453.41 | Acute venous embolism and thrombosis of deep vessels of proximal lower extremity |
| 453.42 | Acute venous embolism and thrombosis of deep vessels of distal lower extremity |
| 453.40 | Acute venous embolism and thrombosis of unspecified deep vessels of lower extremity |
| 453.41 | Acute venous embolism and thrombosis of deep vessels of proximal lower extremity |
| 453.42 | Acute venous embolism and thrombosis of deep vessels of distal lower extremity |
| 453.40 | Acute venous embolism and thrombosis of unspecified deep vessels of lower extremity |
| 453.41 | Acute venous embolism and thrombosis of deep vessels of proximal lower extremity |
| 453.42 | Acute venous embolism and thrombosis of deep vessels of distal lower extremity |
| 453.40 | Acute venous embolism and thrombosis of unspecified deep vessels of lower extremity |
| 453.41 | Acute venous embolism and thrombosis of deep vessels of proximal lower extremity |
| 453.42 | Acute venous embolism and thrombosis of deep vessels of distal lower extremity |
| 453.40 | Acute venous embolism and thrombosis of unspecified deep vessels of lower extremity |
| 453.41 | Acute venous embolism and thrombosis of deep vessels of proximal lower extremity |
| 453.42 | Acute venous embolism and thrombosis of deep vessels of distal lower extremity |
| 453.40 | Acute venous embolism and thrombosis of unspecified deep vessels of lower extremity |
| 453.41 | Acute venous embolism and thrombosis of deep vessels of proximal lower extremity |
| 453.42 | Acute venous embolism and thrombosis of deep vessels of distal lower extremity |
| 453.40 | Acute venous embolism and thrombosis of unspecified deep vessels of lower extremity |
| 453.41 | Acute venous embolism and thrombosis of deep vessels of proximal lower extremity |
| 453.42 | Acute venous embolism and thrombosis of deep vessels of distal lower extremity |
| 453.81 | Acute venous embolism and thrombosis of superficial veins of upper extremity |
| 453.82 | Acute venous embolism and thrombosis of deep veins of upper extremity |
| 453.83 | Acute venous embolism and thrombosis of upper extremity, unspecified |
| 453.84 | Acute venous embolism and thrombosis of axillary veins |
| 453.85 | Acute venous embolism and thrombosis of subclavian veins |
| 453.86 | Acute venous embolism and thrombosis of internal jugular veins |
| 453.87 | Acute venous embolism and thrombosis of other thoracic veins |
| 453.81 | Acute venous embolism and thrombosis of superficial veins of upper extremity |
| 453.82 | Acute venous embolism and thrombosis of deep veins of upper extremity |
| 453.83 | Acute venous embolism and thrombosis of upper extremity, unspecified |
| 453.84 | Acute venous embolism and thrombosis of axillary veins |
| 453.85 | Acute venous embolism and thrombosis of subclavian veins |
| 453.86 | Acute venous embolism and thrombosis of internal jugular veins |
| 453.87 | Acute venous embolism and thrombosis of other thoracic veins |
| 453.89 | Acute venous embolism and thrombosis of other specified veins |
| 453.89 | Acute venous embolism and thrombosis of other specified veins |
| 453.9 | Other venous embolism and thrombosis of unspecified site |
| 453.9 | Other venous embolism and thrombosis of unspecified site |
| 453.9 | Other venous embolism and thrombosis of unspecified site |
| 453.9 | Other venous embolism and thrombosis of unspecified site |
| 453.9 | Other venous embolism and thrombosis of unspecified site |
| 453.9 | Other venous embolism and thrombosis of unspecified site |
| 453.9 | Other venous embolism and thrombosis of unspecified site |
| 453.9 | Other venous embolism and thrombosis of unspecified site |
| 453.9 | Other venous embolism and thrombosis of unspecified site |
| 567.21 | Peritonitis (acute) generalized |
| 136.9 | Unspecified infectious and parasitic diseases |
